# Cognitive frailty and its association with disability among Chinese community-dwelling older adults: a cross-sectional study

**DOI:** 10.1186/s12877-024-04773-0

**Published:** 2024-02-26

**Authors:** Ling-lin Kong, Wen Xie, Zi-yan Dong, Yue-ting Liu, Hui-Min Zhao, Jun-Yao Fan, Xiao-jing Qi, Jie Li

**Affiliations:** 1 School of Public Health and Nursing, Hubei University of Science and Technology, Xianning, China; 2 School of Nursing, Tongji Medical College, Huazhong University of Science and Technology, Wuhan, China; 3 College of Nursing, Shanxi University of Chinese Medicine, Jinzhong, China

**Keywords:** Cognitive frailty, Cognitive impairment, Functional disability, Instrumental activities of daily living, Physical frailty

## Abstract

**Background:**

There are a variety of determinants that are key to functional disability of older adults. However, little is known regarding the relationship between cognitive frailty and disability among older people. The aims of this study were to examine the associations between cognitive frailty and its six components with instrumental activities of daily living (IADL) functioning in community-dwelling older adults.

**Methods:**

A total of 313 community-dwelling older adults (aged ≥ 65 years) were recruited from eight community centers in central China. Cognitive frailty was operationalized using the Mini-Mental State Examination for the evaluation of cognitive status and the Fried criteria for the evaluation of physical frailty. The outcome was functional disability assessed by the IADL scale. The association between cognitive frailty, as well as its components, and IADL limitations was identified by conducting binary logistic regression analysis.

**Results:**

The prevalence of cognitive frailty was 8.9% in this study. The results showed that cognitive frailty (OR = 22.86) and frailty without cognitive impairment (OR = 8.15) were associated with IADL limitations. Subdimensions of cognitive frailty, exhaustion, weakness, low physical activity and cognitive impairment components were independently associated with IADL limitations.

**Conclusion:**

Cognitive frailty was associated with a higher prevalence of disability. Interventions for improving cognitive frailty should be developed to prevent IADL disability among community-dwelling older adults in China.

## Introduction

With the aging of the population in both developed and developing countries, age-related disability has become a public health concern. Disability is mostly defined as experiencing a limitation in activities of daily living (ADL) and/or a limitation in instrumental activities of daily living (IADL) [1], which leads to poorer quality of life, higher healthcare costs and increased mortality [2, 3]. Hence, there is a growing interest in investigating the determinants of disability to relieve societal burden and promote successful aging.

The development of disability in the older population is often complex and a consequence of multiple causes. Numerous research claim that disability is influenced by life-course determinants such as sociodemographic factors [2], lifestyle factors [1], and psychological factors [4]. Recently, preliminary evidence indicates that a new concept of “cognitive frailty” also increases the risks of disability [5]. Cognitive frailty was proposed by an international consensus group in 2013, and it was defined as a heterogeneous clinical manifestation characterized by the coexistence of physical frailty and cognitive impairment without Alzheimer’s disease (AD) or other dementias [6]. There were few studies to explore the relationship between cognitive frailty and ADL/IADL disability [5, 7, 8]. For example, a Singapore study found that compared to robust noncognitive impaired individuals, physical pre-frailty with cognitive impairment was associated with a 2-fold increased prevalence and incidence of functional disability, and cognitively impaired frail individuals stood out with 12- and 13-fold increased prevalence and incidence of functional disability [7]. However, there is little information about the contribution of cognitive frailty to daily functioning among community-dwelling older adults in mainland China.

In addition, the independent contribution of each cognitive frailty component to disability was rarely explored. According to the Fried criteria [9], frailty was defined by the presence of at least three of the five following criteria: unintentional weight loss, muscle weakness, slow walking speed, low physical activity and exhaustion. Therefore, cognitive frailty can be regarded as including six components: the above-mentioned five frailty criteria and cognitive impairment. As six components represent the different physiopathological mechanisms of cognitive frailty, and not all older adults have the same components when being diagnosed as cognitive frailty. Thus, it is necessary to understand the contribution of cognitive frailty components to disability among older adults, so as to develop effective interventions to help older adults delay functional decline.

As IADL decline, which relies on highly complicated neuropsychological organization, generally appears with aging and precedes decline in ADL functioning and dementia [10], we mainly focused on the impact of cognitive frailty on IADL and used IADL measures as an assessment of daily functioning in this study. Therefore, we designed a cross-sectional study to explore the association between frailty and IADL, and our specific aims were as follows: (a) to estimate the prevalence of cognitive frailty; (b) to examine the effect of cognitive frailty on IADL disability; and (c) to identify the independent associations between each of the six cognitive frailty components and IADL disability, in a sample of Chinese community-dwelling older adults.

## Methods

### Study design and setting

A cross-sectional design was employed. The participants were recruited from eight community centers in Wuhan City of Hubei Province in China from September 2018 to May 2019.

### Ethical considerations and data collection

The study was approved by the institutional medical ethics committee (approved No. S941), and written informed consent was obtained from all participants. The data was collected using a self-reported questionnaire in a face-to-face interview administered by three trained research assistants in the communities.

### Participants

Convenience sampling was employed. The inclusion criteria were the following: (a) residents aged ≥ 65 years; (b) inhabitants of Wuhan City without a plan to move shortly; (c) be able to understand and speak Chinese; (d) voluntarily agreed to participate. The exclusion criteria were: (a) with a self-history of diagnosed AD, Parkinson’s disease, or other neurodegenerative disorders; (b) the presence of psychiatric illness; (c) inability to complete all tests due to poor functional status.

### Variables and measurements

#### Operationalization of cognitive frailty

The assessment of cognitive frailty included two parts: physical frailty and cognitive function. The individual with both physical frailty and cognitive impairment would be classified as cognitive frailty.

Physical frailty was assessed based on a modified version of the Fried criteria [9] as follows: (a) Unintentional weight loss was defined as weight loss ≥ 4.5 kg in the past year, not due to dieting and exercise; (b) Slow walking speed was defined, using the average of two-times walking tests (with or without a walking assistant) over a 6 m distance, as ≤ 0.89 m/s for men and ≤ 0.79 m/s for women in normal pace [11]; (c) Weakness was defined, using maximum grip strength of either hand (3 trials for each) measured by a CAMRY electronic dynamometer (Model EH101, Xiangshan Inc, Guangdong, China), as ≤ 28.0 kg for men and ≤ 18.0 kg for women [11]; (d) Self-reported exhaustion was indicated by two questions from the Center for Epidemiological Studies Depression (CES-D) Scale [12]: (i) I felt that everything I did was an effort in the last week; and (ii) I could not get going in the last week. Answers of either “a moderate amount of time (3–4 days)” or “most or all of the time (5–7 days)” met the criteria for exhaustion; and (e) Low physical activity was measured by the Chinese version of Physical Activity Scale for the Elderly (PASE) [13]. Low physical activity was classified by PASE score for men (≤ 56.4) and women (≤ 58.8) [11]. Individuals who met 3–5 criteria were considered “frail”, those who met 1 or 2 criteria were considered “pre-frail”, and those with none of the criteria were defined as “non-frail/robust”.

Cognitive function was assessed using the Mini-Mental State Examination (MMSE) [14]. The MMSE is comprised of five domains: orientation to time and place, memory, attention and calculation, language ability, and recall. The total score ranges from 0 to 30 points, with a lower score indicating poor global cognitive performance. The MMSE has been validated for local use in Chinese older adults, and the education-stratified cut-off points of 16/17 for those who were illiterate, 19/20 for those with 1–6 years of education, and 23/24 for those with more than 6 years of education, was defined as cognitive impairment [15].

The participants were categorized into these six groups: (1) Robust without cognitive impairment; (2) Robust with cognitive impairment; (3) Pre-frail without cognitive impairment; (4) Pre-frail with cognitive impairment; (5) Frail without cognitive impairment; and (6) Frail with cognitive impairment (cognitive frailty group).

### *Measurement of functional disability*

Functional disability was measured using the Instrumental Activities of Daily Living (IADL) Scale, which was developed by Lawton and Brody [16]. The IADL scale consists of 8 items: ability to use telephone, shopping, housekeeping, food preparation, laundry, mode of transportation, responsibility for one’s own medications, and ability to handle finances. Its reliability and validity in the Asian population of community-dwelling older adults have been well established [17]. In this study, functional disability was defined as existence if the participants reported they needed assistance to complete or could not even complete ≥ 1 IADL task.

### *Potential confounding factors of functional disability*

Socio-demographic variables included age, sex, living arrangement (living alone versus living with others), years of education and marital status (married versus single, divorced, widowed or separated). Lifestyles included smoking status (current, former or never smoker), drinking status (current, former or never drinker) and regular exercise (yes or no). Body mass index (BMI) was calculated as weight in kilograms divided by height in square meters. Self-rated health was recorded as poor, fair or good. Participants were asked to report if they had a physician’s diagnosis of one of the following chronic diseases: heart diseases, hypertension, diabetes, cerebrovascular diseases, respiratory diseases, cancer, osteoarthritis, hyperlipemia, kidney diseases, hepatobiliary diseases, peptic ulcer, thyroid diseases, urinary system diseases, cervical and lumbar diseases, vision disorders and hearing disorders. The total number of chronic diseases and regular prescription medications were calculated. Sleep disorders were estimated using dichotomized yes/no responses. Depressive symptoms were determined by the 30-item Geriatric Depression Scale (GDS-30) [18], which has been validated for local use in community-living Chinese older adults [19]. The presence of depressive symptoms was defined as a GDS score of 11 or more.

### Statistical analysis

Statistical analysis was executed by Statistical Package for Social Sciences (SPSS), version 21.0 (SPSS Inc., Chicago, IL, USA). We used Student’s t test and Pearson χ2 test to detect differences in characteristics between participants with and without IADL disability. Binary logistic regression analysis was performed to estimate adjusted odds ratio (OR) and 95% confidence interval (95% CI), and to examine the associations between cognitive frailty and IADL disability. OR were adjusted for potential confounders including age, sex, education, BMI, marital status, living arrangement, chronic diseases, medications, self-rated health, sleep disorders, smoking status, drinking status, regular exercise, cognitive function and depressive symptoms. Binary logistic regression model was also created to evaluate the independent associations between subdimensions of cognitive frailty and IADL limitations after adjusting for the above-mentioned confounders (except for regular exercise and cognitive function). Statistical significance was defined as *P*-value < 0.05.

## Results

A total of 331 older adults consented to participate in this study. Four participants were classified as not eligible due to severe physical impairment, and fourteen participants did not complete the questionnaires due to temporary issues, so the final sample consisted of 313 participants. Of all participants, 80 (25.5%) were robust, 148 (47.3%) were pre-frail, and 85 (27.2%) were frail; and 71 (22.7%) had cognitive impairment. In total, 28 (8.9%) were frail with cognitive impairment (cognitive frailty).

### Characteristics of participants

Table 1 presents the characteristics of the study sample according to IADL limitations. The mean age was 75.7 years (SD = 7.1, range 65–94), 21.1% were male, and 47.3% had primary or lower education. Participants with IADL disability were older, less educated, less likely to be married, had poor self-reported health, had sleep disorders, had less regular exercise, had worse cognitive performance, and had more depressive symptoms compared with those without IADL disability (*P* < 0.05).

**Table 1:** Characteristics of participants (*N* = 313)

	Total (*N* = 313)	Participants without IADL disability (*N* = 181)	Participants with IADL disability (*N* = 132)	*P*-value
Age	75.7 ± 7.1	74.1 ± 6.3	77.8 ± 7.5	< 0.001^a^
Female, n (%)	247 (78.9)	138 (76.2)	109 (82.6)	0.175^b^
Years of education	6.9 ± 4.9	8.0 ± 4.5	5.3 ± 5.0	< 0.001^a^
BMI, kg/m^2^	24.7 ± 3.7	24.9 ± 3.8	24.5 ± 3.6	0.352^a^
Marital status, n (%)				0.010^b^
Married	162 (51.8)	105 (58.0)	57 (43.2)	
Single, divorced, widowed or separated	151 (48.2)	76 (42.0)	75 (56.8)	
Living alone, n (%)	52 (16.6)	30 (16.6)	22 (16.7)	0.983^b^
Number of chronic diseases	4.8 ± 2.9	4.7 ± 2.7	4.9 ± 3.1	0.692^a^
Number of medications	2.3 ± 2.2	2.0 ± 2.1	2.7 ± 2.4	0.009^a^
Self-reported health, n (%)				0.021^b^
Good	175 (55.9)	107 (59.1)	68 (51.5)	
Fair	94 (30.0)	57 (31.5)	37 (28.0)	
Poor	44 (14.1)	17 (9.4)	27 (20.5)	
Sleep disorders, n (%)	183 (58.5)	95 (52.5)	88 (66.7)	0.012^b^
Smoking status, n (%)				0.243^b^
Current	33 (10.5)	23 (12.7)	10 (7.6)	
Former	36 (11.5)	18 (9.9)	18 (13.6)	
Never	244 (78.0)	140 (77.3)	104 (78.8)	
Drinking status, n (%)				0.079^b^
Current	51 (16.3)	35 (19.3)	16 (12.1)	
Former	34 (10.9)	15 (8.3)	19 (14.4)	
Never	228 (72.8)	131 (72.4)	97 (73.5)	
Regular exercise, n (%)	222 (70.9)	138 (76.2)	84 (63.6)	0.015^b^
MMSE score	24.0 ± 4.7	25.9 ± 3.1	21.4 ± 5.4	< 0.001^a^
GDS score	6.5 ± 3.6	5.7 ± 3.4	7.5 ± 3.6	< 0.001^a^

Note: IADL, instrumental activities of daily living; BMI, body mass index; MMSE, mini mental state examination; GDS, geriatric depression scale

^a^ Student’s t test

^b^ Pearson *χ*^*2*^

### Cognitive frailty and IADL disability

To explore the associations between cognitive frailty status and IADL disability, binary logistic regression analysis was computed. The results are presented in Table 2. Compared to being robust without cognitive impairment, being frail without cognitive impairment was significantly more likely to report IADL disability (OR = 8.15, 95% CI = 2.84–23.35). Significant relationship was found between frailty with cognitive impairment (cognitive frailty) and IADL disability (OR = 22.86, 95% CI = 2.09-250.02).

**Table 2:** Associations between cognitive frailty and IADL disability

	IADL disability		
	OR	95% CI	*P-*value
Robust without cognitive impairment (*N* = 68)		1.00 (ref.)	
Robust with cognitive impairment (*N* = 12)	0.63	0.12–3.33	0.588
Pre-frail without cognitive impairment (*N* = 117)	1.40	0.60–3.27	0.442
Pre-frail with cognitive impairment (*N* = 31)	3.34	0.89–12.55	0.075
Frail without cognitive impairment (*N* = 57)	8.15	2.84–23.35	< 0.001
Frail with cognitive impairment (*N* = 28)	22.86	2.09-250.02	0.010

Note: OR: odds ratio; 95% CI: 95% confidence interval; IADL: instrumental activities of daily living

Odds ratio adjusted for age, sex, education, BMI, marital status, living arrangement, chronic diseases, medications, self-rated health, sleep disorders, smoking status, drinking status, regular exercise, cognitive function and depressive symptoms

### Cognitive frailty components and IADL disability

The associations between each component of cognitive frailty and IADL disability are shown in Table 3. Exhaustion, weakness, low physical activity and cognitive impairment components were independently associated with functional limitations after adjustment. In particular, cognitive impairment had the strongest association with IADL disability (OR = 7.72, 95% CI = 3.61–16.53), while weakness had the weakest association with IADL disability (OR = 2.29, 95% CI = 1.18–4.43).

**Table 3:** Associations of cognitive frailty components with IADL disability

	IADL disability		
	OR	95% CI	*P*-value
Weight loss	1.13	0.42–3.04	0.815
Exhaustion	3.23	1.66–6.30	0.001
Slowness	0.97	0.51–1.87	0.937
Weakness	2.29	1.18–4.43	0.014
Low physical activity	2.97	1.52–5.81	< 0.001
Cognitive impairment	7.72	3.61–16.53	< 0.001

Note: OR: odds ratio; 95% CI: 95% confidence interval; IADL: instrumental activities of daily living

Odds ratio adjusted for age, sex, education, BMI, marital status, living arrangement, chronic diseases, medications, self-rated health, sleep disorders, smoking status, drinking status, and depressive symptoms

## Discussion

In this study, we provided an operational definition of cognitive frailty which adopted the Fried criteria for the assessment of physical frailty and the MMSE for cognitive impairment. The prevalence rate of cognitive frailty in our study (8.9%) appeared to be approximately equal to the pooled prevalence in a meta-analysis (9%) [20]. It indicated that cognitive frailty was common among older adults living in the community. A recent study reported that the prevalence of cognitive frailty among community-dwelling older adults in Spain was 23.61% [21]. Another Chinese research reported that the prevalence of cognitive frailty was 11.8% among older adults in Western China [22]. The difference in the prevalence of cognitive frailty may be attributable to the different operational definition of the two components of cognitive frailty (physical frailty and cognitive function) and different investigated regions and populations. Notably, the prevalence of cognitive frailty has increased in recent years. A meta-analysis reported that the pooled estimates of cognitive frailty prevalence were 6% from 2012 to 2017 and 11% from 2018 to 2020 [20]. Thus, the cognitive frailty of older adults is an area of concern.

The relationship between frailty and IADL disability has been universally proven [23]. The result of our study that the older adults were frail without cognitive impairment (physical frailty) had an association with a higher prevalence of IADL limitation was consistent with the previous study [24]. In addition, we also found that frail older adults with cognitive impairment (cognitive frailty) had an association with a higher prevalence of IADL disability which was the same as the results of another study [25]. It’s worth noting that we found that the relationship (OR = 22.86) between cognitive frailty and IADL disability was stronger than the relationship (OR = 8.15) between frailty and IADL disability. This finding in our study was consistent with a previous study, which showed that relative to participants who had normal cognition and were non-frail, those who were frail with cognitive impairment (OR = 4.47) and frail with normal cognition (OR = 2.62) had a higher prevalence of IADL [24]. Cognitive frailty was associated with a higher prevalence of IADL disability due to the accumulation of adverse outcomes which was caused by the simultaneous presence of both physical frailty and cognitive impairment [25]. This finding may imply that compared to physical frailty, cognitive frailty is more sensitive to capturing older adults who are at high risk of developing IADL disability and it could be a better measure of vulnerability for community-dwelling older adults [24]. However, the cognitive frailty of the older adults in the community is usually ignored [26]. We should attach great importance to the early identification of cognitive frailty among community-dwelling older adults in the future.

Our results showed that in subdimensions of cognitive frailty, the cognitive impairment component had an association with a higher prevalence of IADL disability. Previous studies also demonstrated a strong association between cognitive impairment and subsequent functional declines [5, 27]. A systematic review including 37 studies reported that compared to subjects with normal cognition, those with MCI spent more time completing IADL tasks and tended to be less accurate, especially in more complex tasks requiring higher cognitive processes [27]. As IADL involves a series of complex daily activities, such as grocery shopping, management of finances or taking medicine, that highly depend on the integrity of neuropsychological functioning, the effect of cognitive impairment on IADL disability may be easily understood [28]. This finding suggests that cognitive impairment could be considered as an early indicator of impending functional declines for older people. Therefore, we need to pay more attention to the cognitive function of old adults and commit to developing effective interventions to improve cognitive function, so as to prevent IADL disability of older adults.

In agreement with findings in population-based studies [29–30], our results indicated that low physical activity was a contributor to functional disability in older adults. A Mexican cross-sectional study showed that low physical activity was significantly associated with ADL and IADL limitations after adjustment for covariates [29]. Another research also found that physical inactivity was a factor strongly associated with ADL and IADL functional limitations in older Korean adults [30]. The contribution of physical activity to functional impairment may be mediated by a positive effect on muscle strength [31]. Another supposed mechanism is related to reduced inflammation biomarkers, such as C-reactive protein (CRP) and interleukin-6 (IL-6) [32]. Although the underlying biological mechanisms remain unclear, it still suggests that physical activity could be a potential target for compensatory and interventive strategies to prevent functional declines and disability among older adults in the community.

The current study also revealed that self-reported exhaustion was independently associated with IADL limitations, consistent with the findings reported by Gobbens and van Assen who found that fatigue was associated with ADL and IADL disability of community-dwelling older adults in the Netherlands [33]. In contrast, a Mexican study investigated the association of six components of cognitive frailty (including five criteria from the Fried phenotype and cognitive impairment) with prevalent functional disability, and found that the exhaustion component was not associated with any type of disability after adjusting for potential confounders [29]. Rothman et al. also reported that self-reported exhaustion was initially associated with chronic disability after adjustment for socio-demographic factors and comorbidities, but the association was no longer statistically significant after adding the other frailty criteria as covariates [34]. These discrepant findings may be due to differences in the populations included, functional disability defined or exhaustion measures used. The absence of a correlation between exhaustion and physical functioning in the previous research suggests that exhaustion may not be a good predictor of functional disability. Further research is needed to confirm the effect of exhaustion on physical functioning in old age, especially in community-dwelling older adults.

A relationship between muscle weakness and IADL disability was identified in our study, indicating that lower grip strength may adversely affect functional ability. These findings are in accordance with an earlier study which found a strong relationship between low handgrip strength and incident disability in a large sample of Japanese adults ≥ 65 years old [35]. McGrath et al. reported that greater muscle strength was related to decreased odds of 2-year onset of ADL and IADL disability in older Mexican Americans [36]. During the aging process, the decline in skeletal muscle mass and an increase in fat mass may happen in older adults [37], muscle strength may decrease to a certain degree where weakness begins to restrict the ability to carry out daily activities, such as cooking, housework, and laundry. Hence, these results highlight the importance of maintaining muscle strength in preserving functional independence in older age. It is recommended that intervention programs such as resistance exercises should be carried out to enhance muscle strength among older adults in the community.

This study provides empirical evidence about the association between cognitive frailty and functional disability in mainland China; moreover, this data is essential for reporting the independent contribution of the six cognitive frailty components to IADL performance in a sample of Asian community-dwelling older population. Based on the most common criteria for the assessment of physical frailty (the Fried criteria) and the worldwide valid and reliable instrument for the evaluation of cognitive declines (MMSE), our operational definition could be a feasible screening tool for cognitive frailty and may be adopted in future research. In addition, we also included a wide range of covariates related to IADL limitations.

However, there are several limitations in this study. First, as the design of the research was cross-sectional, it is impossible to establish causality or determine the direction of the association between cognitive frailty and IADL decline. The second limitation concerns that participants were not recruited randomly and the sample size is relatively small, which might have biased our results and made it difficult to generalize these findings to the whole Chinese older population. Third, information bias related to miscommunication, recall error, cognitive status and other inherent misrepresentations may happen as the data were collected based on a self-reported questionnaire. Finally, the adoption of slightly different criteria to define physical frailty, which potentially limited the comparability with results from other studies. Thus, further studies with larger sample size and longitudinal study design are needed to identify the predictive value of cognitive frailty on disability among older adults.

## Conclusions

Cognitive frailty found in 8.9% of this community-dwelling older adults was associated with the highest risk of IADL limitations. Moreover, frail individuals with or without cognitive impairment had an association with a higher prevalence of functional declines than robust older adults with normal cognition. Subdimensions of cognitive frailty, exhaustion, weakness, low physical activity and cognitive impairment components were associated with IADL disability. The findings of this study could help guide future studies to implement targeted and suitable interventions for preventing IADL disability among community-dwelling older adults. Furthermore, longitudinal studies with larger samples are necessary to better inspect the predictive value of cognitive frailty on disability in older adults.

## Data Availability

The datasets used and analyzed during the current study are available from the corresponding author on reasonable request.

## Abbreviations

IADL: Instrumental activities of daily living
ADL: Activities of daily living
AD: Alzheimer’s disease
CES-D: Center for Epidemiological Studies Depression Scale
PASE: Physical Activity Scale for the Elderly
MMSE: Mini-Mental State Examination
GDS-30: 30-item Geriatric Depression Scale
SPSS: Statistical Package for Social Sciences
OR: Odds ratio
CI: Confidence interval
SD: Standard deviation
BMI: Body mass index
SLAS: Singapore Longitudinal Ageing Studies
MCI: Mild cognitive impairment
CRP: C-reactive protein
IL-6: Interleukin-6
